# Clinical outcomes of posttransplantation diabetes mellitus in kidney transplantation recipients: a nationwide population-based cohort study in Korea

**DOI:** 10.1038/s41598-022-25070-z

**Published:** 2022-12-14

**Authors:** Eunjeong Kang, Jangwook Lee, Dong Hyun Kang, Jina Park, Sehoon Park, Yong Chul Kim, Dong Ki Kim, Kwon Wook Joo, Yon Su Kim, Minsu Park, Yaeji Lim, Hajeong Lee

**Affiliations:** 1 Department of Internal Medicine, Ewha Womans University Seoul Hospital, Ewha Womans University College of Medicine, Seoul, Korea; 2 Department of Internal Medicine, Dongguk University Ilsan Hospital, Goyang, Korea; 3 Department of Applied Statistics, Chung-Ang University, 84 Heukseok-Ro, Dongjak-Gu, Seoul, 06974 Korea; 4 Biomedical Research Institute, Seoul National University Hospital, Seoul, Korea; 5 Department of Biomedical Sciences, Seoul National University College of Medicine, Seoul, Korea; 6 Department of Internal Medicine, Seoul National University Hospital, 101 Daehak-Ro, Jongno-Gu, Seoul, 03080 Korea; 7 Department of Internal Medicine, Seoul National University College of Medicine, Seoul, Korea; 8 Department of Informations and Statistics, Chungnam National University, Daejeon, Korea

**Keywords:** Nephrology, Kidney

## Abstract

Posttransplantation diabetes mellitus (PTDM) is an important metabolic complication after KT that causes graft failure and cardiovascular complications in kidney transplantation (KT) recipients. Using the national claim data of South Korea, 7612 KT recipients between 2009 and 2017 were analyzed. PTDM was defined as a consecutive 30-day prescription history of antidiabetic medication after KT. Among these patients, 24.7% were diagnosed with PTDM, and 51.9% were diagnosed within 6 months after KT. Compared to patients without PTDM, those with PTDM were older, more likely to be men, more likely to be diagnosed with hypertension and cardio-cerebrovascular disease, and experienced more rejection episodes requiring high-dose steroid treatment after KT. During the follow-up, 607 DCGFs, 230 DWGFs, 244 MACEs, and 260 all-cause mortality events occurred. Patients with PTDM showed a higher risk of DCGF (adjusted hazard ratio [aHR] 1.49; 95% confidence interval [CI] 1.22–1.82; *P* < 0.001) and MACEs (aHR 1.76; 95% CI 1.33–2.31; *P* < 0.001) than patients without PTDM. The risks for all clinical outcomes were higher in the insulin group than in the non-use insulin group. PTDM in KT recipients resulted in both worse allograft and patient outcomes represented by DCGF and MACE, especially in patients needing insulin treatment.

## Introduction

The outcomes of kidney transplants (KTs) have improved over the previous decade, although numerous medical complications emerging after KT may have a long-term impact on recipients’ health and quality of life. Posttransplantation diabetes mellitus (PTDM) is one of the most important complications in KT recipients^1^. The incidence of PTDM varies from 4 to 25%, which is likely due to the lack of a standard definition of PTDM^2^.

KT recipients with PTDM demonstrated complications similar to those exhibited by patients with type 2 diabetes mellitus (DM), including an increased incidence of cardiovascular and infectious events and all-cause mortality^3–7^, but at an accelerated rate^8^. In addition, according to the United States Renal Data System data, KT recipients with PTDM were at a higher risk of death-censored graft failure (DCGF) and all-cause death than those who did not develop PTDM. With the increasing number of KT recipients with extended survival, it is essential to identify and monitor the risk factors for PTDM in KT recipients^9^. Numerous risk factors have been reported for predicting PTDM independently. Modifiable risk factors for PTDM include obesity, metabolic syndrome, immunosuppressive agents, hypomagnesemia, decreased physical activity, and viral infections, including hepatitis C and cytomegalovirus^9–13^; non-modifiable risk factors known so far include age, family history of DM, male sex, genetic polymorphism, deceased donor, and increased number of HLA mismatches^9,11,14,15^. Ethnicity is one of the crucial non-modifiable risk factors for PTDM; however, the evidence on ethnicity risk for PTDM has primarily concentrated on data pertaining to African Americans and Hispanics^16,17^. South Korea is one of the developed countries dealing with a population-aging issue, which is attributed to a rapidly increasing prevalence of end-stage renal disease^18^. Accordingly, the number of transplantation cases in Brazil has been steadily increasing since the initial kidney transplantation cases in 1969^19^. All-cause mortality has improved recently despite the fact that high-risk transplant cases have become increasingly common^19^. This suggests that the incidence of PTDM, which is a long-term complication of KT, will also increase. However, detailed information on the epidemiology and prognosis of PTDM in Koreans is scarce.

Moreover, as aforementioned, many studies have shown that PTDM is associated with poor clinical outcomes in KT recipients^9,20–22^. To improve the clinical outcomes of KT recipients, it is important to investigate which characteristics among KT recipients diagnosed with PTDM may be associated with worse outcomes. Therefore, we aimed to investigate the nationwide epidemiology of PTDM in South Korea. Furthermore, we sought to assess the clinical outcomes of PTDM in Korean KT recipients and create valuable evidence that can be used to influence future strategies for treating PTDM.

## Materials and methods

### Ethical considerations

This study was approved by the Institutional Review Board (IRB) of the Seoul National University Hospital (IRB number [no.]: E-2103-137-1206) and the need for informed consent was waived by this IRB. This study was conducted in accordance with the principles of the Declaration of Helsinki. The Health Insurance and Review Assessment (HIRA) service approved the database analysis (no.: M20210324185).

### Study design, setting, and cohort

**Figure 1:**
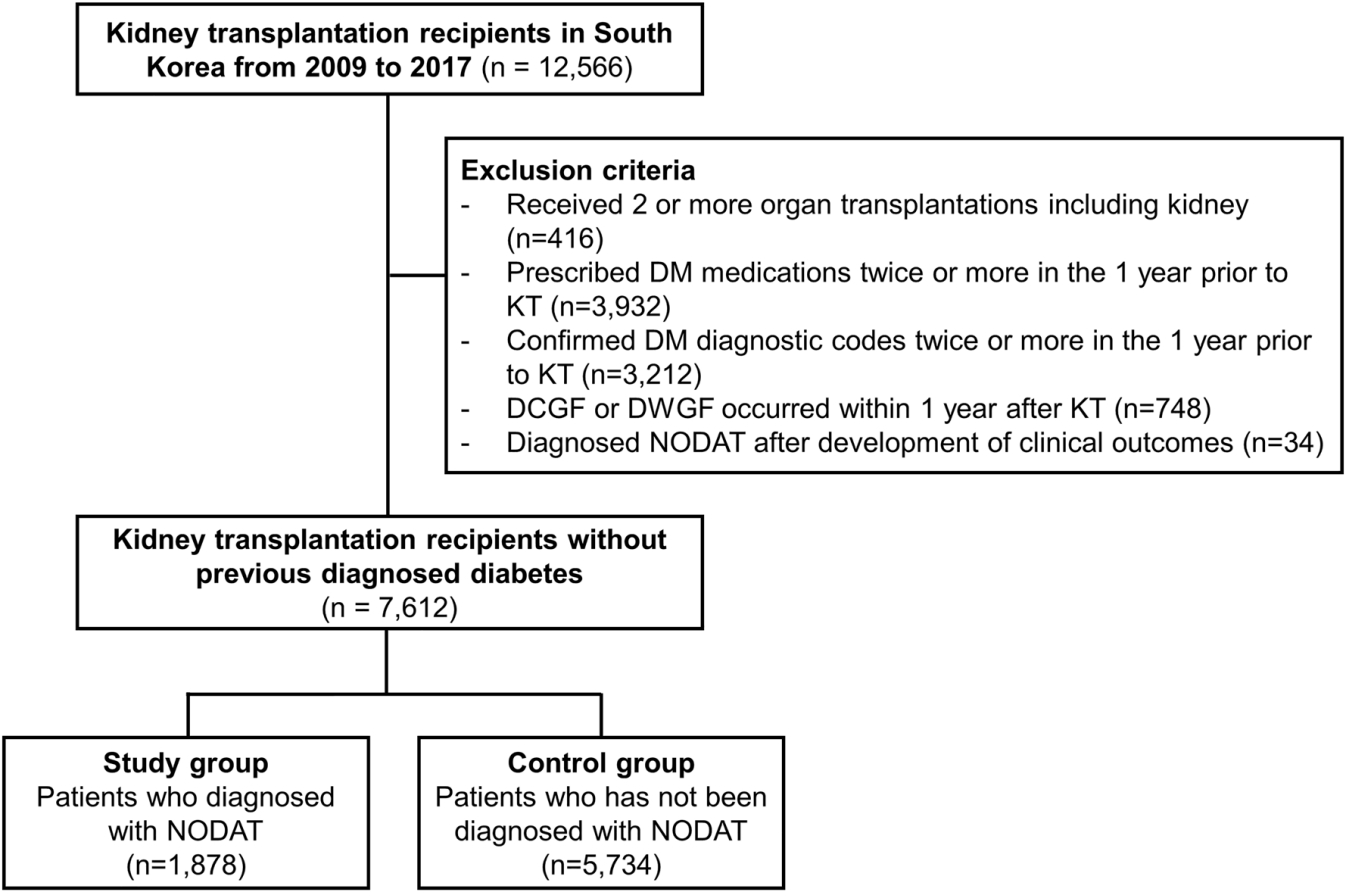
Flow chart of the study. *DM* diabetes mellitus, *KT* kidney transplantation, *PTDM* new-onset diabetes after kidney transplantation.

The National Health Insurance Service (NHIS) of Korea enforces legal obligation and authority as a universal social insurance program that covers the entire South Korean population. The HIRA operates under the NHIS and evaluates healthcare costs and quality, supports medical policy and reviews, and authorizes claims issued regarding insured medical services. The claims database maintained by HIRA has been used in many epidemiological studies. Given that KT surgery is an insured medical service, we identified KT recipients and their characteristics by reviewing the HIRA database. In this nationwide, retrospective cohort study, we included all KT recipients from 2009 to 2017 who were identified by the specific codes for KT (R3280 [KT, International Classification of Diseases, Tenth Revision {ICD-10} code] and V005 [KT-related treatment, V code for rare incurable Korean diseases]). The exclusion criteria were as follows: (1) patients who received ≥ 2 organ transplantations including the kidney; (2) those who were prescribed diabetes medications ≥ 2 times in the year prior to KT; (3) those with confirmed diagnostic codes for diabetes (E109, E119, E139, E149, E101, E111, E131, E141, E105, E115, E135, and E145) ≥ 2 times in the 1 year prior to KT; (4) patients in whom DCGF and death with graft function (DWGF) occurred within 1 year after KT; and (5) patients who were diagnosed with PTDM after the development of clinical outcomes (Fig. 1).

PTDM was defined as the prescription of antidiabetic medications for ≥ 30 consecutive days after KT. Patients diagnosed with PTDM were included in the study. The control group included patients who were not diagnosed with PTDM.

### Data collection

We collected the following baseline characteristics of the KT recipients: age; sex; previous main dialysis modality; previous comorbidities including hypertension, DM, and dyslipidemia; and induction and maintenance immunosuppressants. Preemptive KT was defined as KT performed without dialysis or dialysis for < 3 months. Patients’ medical history including underlying comorbidities was reviewed in the prior 1 year using ICD-10 codes, prescribed drug records, and the presence of underlying hypertension and dyslipidemia. Records of induction therapy, desensitization, and maintenance immunosuppressive agent use were determined by reviewing the claims database after the KT date. Information on antidiabetic medications was collected: α-glucosidase inhibitors, sulfonylurea, biguanide, thiazolidinedione, meglitinide, dipeptidyl peptidase-4 inhibitors, sodium-glucose cotransporter-2 inhibitors, glucagon-like peptide agonists, and insulin.

### Study outcomes

Patient follow-up was conducted until the censoring of the claims data or July 31, 2020. We evaluated adverse kidney and patient outcomes such as all-cause mortality, major adverse cardiovascular events (MACEs), graft failure, and DWGF. As the direct death date was not recorded in the HIRA database, we used the absence of any claims for > 1 year as an operative criterion for mortality^19,23^. MACE was defined as a composite of acute myocardial infarction, coronary revascularization, and acute ischemic stroke, based on definitions in prior studies that used the claims database^24,25^. DCGF was determined by re-initiation of maintenance dialysis after KT, and the first date of successive dialysis session for 3 months was the outcome. DWGF was defined as the death of KT recipients who had retained kidney function without the need for dialysis or re-transplantation. DWGF was defined as exclusion of DCGF cases from the events of all-cause mortality.

### Statistical analyses

For continuous variables, baseline characteristics are described using mean ± standard deviation or median (interquartile range) according to normal or non-normal distribution, as appropriate. Frequency is expressed as percentage for categorical variables. The Student t-test was used to compare continuous variables between the groups, and Fisher exact test or chi-square test was used to compare categorical variables, as appropriate.

We examined the effect of PTDM on outcomes using Cox proportional hazards analysis, with PTDM modelled as a time-dependent covariate. Age, sex, history of hypertension and dyslipidemia, dialysis modality, dialysis duration, induction therapy, desensitization, transplant rejection, and steroid use were included as covariates in the multivariable models. In the case of a patient who died without MACE was considered as a competing risk for MACE, thus further analysis was carried out using the Fine and Gray model for MACE event.

The patients were further evaluated according to subgroups segregated by the control of DM or not. Since the NHIS and HIRA data did not include laboratory results such as hemoglobin A1c and fasting glucose values, the control of DM was defined based on the use of insulin, number of tablets of DM medications (1 tablet, 2 tablets, and ≥ 3 tablets), whether DM medications were stopped, and the time of diagnosis with PTDM (< 0.5, 0.5 to < 2 years, and ≥ 2 years). The subgroup that stopped prescribed DM medications after the diagnosis of PTDM was defined as those who stopped prescribed medications for > 1 year after the diagnosis of PTDM. We examined the effect of cessation of DM medication in time-dependent Cox models. Other variables were analyzed using the Cox proportional hazards model.

To compensate for differences between groups with and without PTDM, sensitivity analyses were performed with propensity score matching with age, sex, history of hypertension, dyslipidemia, dialysis modality and duration as matching variables.

Statistical analyses were performed using R (version 3.5.1; The R Foundation for Statistical Computing, Vienna, Austria, http://www.R-project.org). A two-sided *P*-value of < 0.05 was considered statistically significant.

## Results

### Baseline characteristics of KT recipients according to the presence of PTDM

Among a total of 12,566 KT recipients, those who received ≥ 2 organ transplantations including the kidney (n = 416), those who were prescribed antidiabetic medications ≥ 2 times in the year prior to KT (n = 3,932), those who had confirmed DM diagnostic codes ≥ 2 times in the year prior to KT (n = 3,212), those who were diagnosed with DCGF or DWGF within 1 year after KT (n = 748), and patients who were diagnosed with PTDM after the development of clinical outcomes (n = 34) were excluded. Finally, a total of 7,612 patients were included in this analysis (Fig. 1).

Figure S1 showed the proportion of prescribed diabetes medication from 6 months before and 6 months after kidney transplantation. Immediately after transplantation, the use of insulin was increased up to around 60%, and then the prescription of biguanides gradually increased after transplantation.

Of 7612 participants, 1878 (24.7%) recipients were diagnosed with PTDM at a median follow-up of 7.0 (interquartile range 4.7–9.2) years. These patients were analyzed for baseline characteristics according to PTDM (Table 1). The median time to diagnosis of PTDM was 5.1 (interquartile range 1.4–34.3) months after KT. Among recipients with PTDM, 51.9% were diagnosed within 6 months after KT, 17.9% between 6 and 24 months, and 30.2% after 24 months after KT. The median age and percentage of male patients were higher in the PTDM group than in the non-PTDM group. Patients diagnosed with dyslipidemia and cardiovascular disease were more common in the PTDM group than in the non-PTDM group. The dialysis type and duration as a continuous variable; prescribed maintenance immunosuppressive agents including calcineurin inhibitors, antimetabolites, and steroids; and medical history of hypertension did not exhibit significant differences between the groups. However, KT recipients with PTDM used more high-dose steroid treatment than their counterparts, which may be associated with acute rejection episodes.

**Table 1:** Baseline characteristics of total enrolled participants according to PTDM.

Variables	PTDM (−) (n = 5734)	PTDM (+) (n = 1878)	*P*
**Age, years**	42.7 ± 12.6	49.6 ± 10.7	< 0.001
≤ 30 years	964 (16.8%)	97 (5.2%)	< 0.001
31–40 years	1375 (24.0%)	256 (13.6%)	
41–50 years	1714 (29.9%)	560 (29.8%)	
51–60 years	1321 (23.0%)	694 (37.0%)	
> 60 years	360 (6.3%)	271 (14.4%)	
**Gender, male, n (%)**	3163 (55.2%)	1120 (59.6%)	0.001
**Dialysis modality**			0.359
Hemodialysis	2589 (45.2%)	885 (47.1%)	
Peritoneal dialysis	1043 (18.2%)	315 (16.8%)	
Mixed	277 (4.8%)	95 (5.1%)	
Preemptive	1825 (31.8%)	583 (31.0%)	
**Dialysis duration (years)**	2.6 ± 2.5	2.7 ± 2.5	0.521
None	627 (10.9%)	186 (9.9%)	0.003
< 1 years	1838 (32.1%)	540 (28.8%)	
1 years–2 years	732 (12.8%)	271 (14.4%)	
2 years–3 years	586 (10.2%)	234 (12.5%)	
≥ 3 years	1951 (34.0%)	647 (34.5%)	
**Underlying disease**			
Hypertension	5449 (95.0%)	1799 (95.8%)	0.199
Dyslipidemia	2841 (49.5%)	1064 (56.7%)	< 0.001
Cardiovascular disease	802 (14.0%)	383 (20.4%)	< 0.001
**Induction therapy**			< 0.001
None	194 (3.4%)	36 (1.9%)	
Thymoglobulin	627 (10.9%)	180 (9.6%)	
Basiliximab	4742 (82.7%)	1583 (84.3%)	
Both	171 (3.0%)	79 (4.2%)	
**Maintenance immunosuppression**			
Tacrolimus	4229 (73.8%)	1344 (71.6%)	0.068
Cyclosporine	500 (8.7%)	152 (8.1%)	0.427
MMF	3848 (67.1%)	1220 (65.0%)	0.092
Steroid	4372 (76.2%)	1421 (75.7%)	0.630
**Rejection**	697 (12.2%)	265 (14.1%)	0.030
High dose steroid	642 (11.2%)	238 (12.7%)	0.043

*PTDM* New onset diabetes after kidney transplantation, *MMF* mycophenolate mofetil.

### Association between PTDM and the risk of adverse kidney and patient outcomes

The median follow-up durations were 78.5 months (49,904.4 person-years in total) for DCGF, 83.1 months (51,962.4 person-years in total) for DWGF, 81.6 months (51,181.1 person-years in total) for MACEs, and 83.1 months (51,962.4 person-years in total) for mortality. The 7612 transplantations resulted in 607 instances of DCGF, 230 instances of DWGF, 244 instances of MACEs, and 260 instances of all-cause mortality. Table 2 shows the association between PTDM and the risk of adverse clinical outcomes. Compared to KT recipients without PTDM, those with PTDM had higher rates of DCGF (adjusted hazard ratio [aHR] 1.49; 95% confidence interval [CI] 1.22–1.82; *P* < 0.001) and MACEs (aHR 1.70; 95% CI 1.29–2.25;* P* < 0.001). There is no difference between the results using a time-dependent cox analysis and competing risk analysis (aHR 1.79; 95% CI 1.36–2.35; *P* < 0.001) for the MACE outcome. Among the KT recipients in this study, PTDM was not associated with DWGF or all-cause mortality.

**Table 2:** Incidence rate and hazard ratio of adverse outcomes according to PTDM.

Outcomes	PTDM	N	Cases	Person-years	Incidence rate	Model 1		Model 2		Model 3	
						HR (95% CI)	*P*	HR (95% CI)	*P*	HR (95% CI)	*P*
**DCGF**											
	PTDM (−)	5734	453	37,271.3	12.2	Reference		Reference		Reference	
	PTDM (+)	1878	154	12,633.1	12.2	1.32 (1.09–1.59)	0.004	1.51 (1.23–1.84)	< 0.001	1.49 (1.22–1.82)	< 0.001
**DWGF**											
	PTDM (−)	5734	166	38,791.4	4.3	Reference		Reference		Reference	
	PTDM (+)	1878	64	13,171	4.9	1.57 (1.17–2.10)	0.003	1.12 (0.84–1.51)	0.443	1.11 (0.83–1.49)	0.489
**MACE**											
	PTDM (−)	5734	153	38,272	4.0	Reference		Reference		Reference	
	PTDM (+)	1878	91	12,909.1	7.1	2.59 (1.99–3.37)	< 0.001	1.69 (1.28–2.23)	< 0.001	1.70 (1.29–2.25)	< 0.001
**All-cause mortality**											
	PTDM (−)	5734	187	38,791.4	4.8	Reference		Reference		Reference	
	PTDM (+)	1878	73	13,171	5.5	1.57 (1.19–2.06)	0.001	1.12 (0.85–1.48)	0.417	1.11 (0.84–1.47)	0.449

Model 1: Univariate analysis.

Model 2: Model 1 + adjustment with age, sex, history of hypertension, dyslipidemia, and cardiovascular disease, dialysis modality, duration of dialysis.

Model 3: Model 2 + adjustment with induction therapy, desensitization, rejection, use of steroid.

*PTDM* New onset diabetes after kidney transplantation, *N* number, *HR* hazard ratio, *CI* confidence interval, *DCGF* death-censored graft failure, *DWGF* death with graft function, *MACE* major adverse cardiovascular events.

### Subgroup analyses in KT recipients with PTDM

Then, we tried to identify the risk factors associated with worse clinical outcomes among KT recipients experiencing PTDM, and the results are shown in Table 3 and Tables S1–3. First, we explored the effect of severity or degree of glucose control on adverse outcomes. Because our database could not use laboratory test results such as hemoglobin A1c or fasting glucose levels, we considered the use of insulin or the number of oral antidiabetic drugs to indicate the severity of PTDM. As expected, insulin users showed higher risks of all clinical outcomes than insulin non-users in the Cox regression analysis (Table 3). Second, we compared the patient and allograft outcomes among insulin non-users according to the number of concomitant oral antidiabetic medications (Table S1). No statistically significant differences were observed in any outcome between the group that used more oral hypoglycemic agents and the group that used fewer medications. Next, we explored the effect of the time to diagnosis of PTDM on the risk of outcomes. All the clinical outcomes were not different between the groups (Table S2). Finally, we examined whether the newly developed hyperglycemia after KT was maintained by following the antidiabetic drug prescription periods. In total, 516 (27%) patients with PTDM discontinued their antidiabetic treatment during the 4.9 (interquartile range, 2.7–7.4) years of follow-up. The discontinuation of antidiabetic drugs was not associated with patient or kidney outcomes, except DWGF (Table S3). Patients who had PTDM but discontinued their antidiabetic treatment showed a lower risk of DWGF than those taking their medications (aHR 0.42; 95% CI 0.21–0.87, *P* = 0.019).

**Table 3:** Subgroup analyses in KT recipients according to the use of insulin.

Outcomes	Model 1		Model 2		Model 3	
	HR (95% CI)	*P*	HR (95% CI)	*P*	HR (95% CI)	*P*
**DCGF**						
No insulin	Reference		Reference		Reference	
Use insulin	2.72 (1.89–3.91)	< 0.001	2.74 (1.90–3.95)	< 0.001	2.72 (1.88–3.93)	< 0.001
**DWGF**						
No insulin	Reference		Reference		Reference	
Use insulin	4.36 (2.28–8.36)	< 0.001	3.96 (2.06–7.60)	< 0.001	3.76 (1.95–7.26)	< 0.001
**MACE**						
No insulin	Reference		Reference		Reference	
Use insulin	2.02 (1.29–3.15)	0.002	2,05 (1.31–3.22)	0.002	2.10 (1.33–3.29)	0.001
**All-cause mortality**						
No insulin	Reference		Reference		Reference	
Use insulin	4.10 (2.25–7.48)	< 0.001	3.75 (2.05–6.85)	< 0.001	3.53 (1.92–6.48)	< 0.001

Model 1: Univariate analysis.

Model 2: Model 1 + adjustment with age, sex, history of hypertension, dyslipidemia, and cardiovascular disease, dialysis modality, duration of dialysis.

Model 3: Model 2 + adjustment with induction therapy, desensitization, rejection, use of steroid.

*HR* hazard ratio, *CI* confidence interval, *DCGF* death-censored graft failure, *DWGF* death with graft function, *MACE* major adverse cardiovascular events.

### Sensitivity analyses using propensity score matching

Propensity score matching was carried out with sensitivity analyses to adjust variables that can affect various clinical outcomes and the results were showed in the Tables S4 and S5. Respectively, 1878 individuals were assigned to each group with or without PTDM. After matching, there was no statistically significant differences between the two groups in age, sex, dialysis duration, history of dyslipidemia, and cardiovascular disease (Table S4). Similar to before propensity score matching, the hazard ratio was significant, found to be > 1.0 in the group with the development of PTDM in DCGF (HR 1.83, 95% CI 1.42–2.35) and MACE (HR 1.79, 95% CI 1.30–2.46).

## Discussion

Post transplantation diabetes mellitus (PTDM) is related to the use of immunosuppressive agents after transplantation and is known to be associated with increased morbidity and mortality in transplant patients. In this study, the term PTDM was used to focus more on newly developed diabetes after transplantation. PTDM is an important metabolic complication after KT that causes graft failure and cardiovascular complications in KT recipients. Regarding the negative impact of PTDM on graft function, this study’s results suggest that PTDM is related to several unfavorable graft and patient outcomes in KT recipients. The detrimental effects of PTDM on DCGF and MACEs were significant even after time-varying Cox analyses in this nationwide cohort study in South Korea. In addition, in subgroup analyses, a poor prognosis was observed in KT recipients who were prescribed insulin.

Diabetes develops in two distinct phases after KT; recipients are initially at the greatest risk within the first 6 months after the transplant, and the number of recipients with diabetes increases gradually over time thereafter^3,26^. In this study, the proportion of those diagnosed with PTDM within 6 months after KT was the highest at 51.8%. In other studies, PTDM occurred in up to 26% of KT recipients in the first 6 months posttransplant^27^, with an annual incidence of 6%^28^. Hsuan et al. reported the highest incidence of PTDM within the first year after KT in a population-based study^20^. In the most recent study, it was reported that 63% of patients were diagnosed with PTDM within 12 months after KT during the median follow-up of 4 years^29^; however, the diagnostic criteria and operational definition were different for each study, making it difficult to accurately compare those study findings with ours.

Glucose metabolism after KT is a dynamic and complicated process, and disorders of glucose homeostasis in KT recipients constitute a significant concern^16^. Hyperglycemia contributes to an increased risk of vascular disease burden, especially in KT recipients who already have vascular risk factors or had previous cardiovascular events. Since patients with PTDM and those with pretransplant diabetes have comparable metabolic and cardiovascular risk factors, the mechanisms for cardiovascular outcomes may be similar. Several population-based cohort studies have shown the relationship between PTDM and the risk of posttransplant MACEs, cardiac and all-cause death, and poor quality of life^6,30^. To date, most studies have mainly focused on Western populations, and only a few studies have been reported in the Asian population. In Japan, the incidence rate of PTDM was 15.1% at 5 years^31^. Additionally, Yuka et al.’s study showed that graft loss was significantly higher in KT recipients with diabetes than in those without during the median 105.5-month follow-up period at a single institution^32^. In a Taiwanese population-based study, PTDM was related to MACEs and patient survival, especially in those who were comparatively young and had fewer comorbidities^20^. However, Malik et al.’s study showed no association between PTDM and graft failure results^29^, we thought that this difference could be explained by shorter follow-up period after KT compared to the present study. Interestingly, unlike most other previous studies, the association between PTDM and total mortality was not confirmed in the present study. We thought that the disparity in mortality of PTDM patient was due to the possibility of enrolling healthy patients who were temporarily prescribed antidiabetic medication by using the operational definition of PTDM. There are still controversies regarding the clinical outcomes of PTDM; further studies are needed to clarify the prognosis of PTDM and to fill these gaps between studies.

In the present study, MACEs were significantly associated with PTDM (Table 2), whereas there was no statistically significant association with all-cause mortality. Owing to the limitations of this claim data, the cause of death could not be accurately determined. In a previous study^33^, the incidence of posttransplant death caused by cardiovascular disease showed a greater decrement over the decades. This study analyzed data from the recent 2000s when cardiovascular death was low. In addition, even if MACEs occurred more frequently in patients with PTDM than in those without, it is likely that MACEs developed as a non-fatal cardiovascular outcome, in other words, not reaching death. Additionally, it is highly likely that there was no difference in all-cause mortality as a cause of death, such as cancer and infection, which are thought to be less related to PTDM. In the subgroup analyses of patients diagnosed with PTDM, the risks of all clinical adverse outcomes increased in insulin users compared to insulin non-users (Table 3). Maintaining proper glucose levels with insulin rather than oral antidiabetic medications means that it is highly likely that glucose is not well controlled. As there have already been many reports that high blood glucose levels are related to cardiovascular events^6,34^, adverse graft outcomes^35,36^, and infection^9^, the present study’s results can be understood in the same context.

Additionally, emerging antidiabetic drugs such as SGLT2 inhibitors and GLP1 receptor agonists are beneficial in reducing cardiovascular events, mortality, and event renal progression. However, there have been limited data in kidney transplantation recipients. Also, since this study used claim data, there is a limitation that we could not identify the drugs prescribed as uninsured. GLP-1 agonists and SGLT-2 inhibitors became available for insurance claims for the first time in 2010 and 2014, respectively. Both drugs were challenging for all diabetic patients due to strict insurance criteria in Korea immediately after insurance. Partly, our recent study based on electronic medical record review may provide some evidence to clarify the role of SGLT2 inhibitors in KT recipients^37^.

This nationwide, retrospective cohort study was performed to investigate the effect of PTDM on patient- and allograft-related outcomes in KT recipients. However, some limitations of this study should be noted. First, because this study was conducted based on a national claims database that has innately limited sensitivity, there might have been inaccessible medical information outside the nation or imprecise insurance codes. In addition, specific variables, including donor information, cause of death, non-insured medical costs, such as desensitization therapy, and actual cause of death, were not included in this study. Especially, competing risk analyses should be considered because MACE and death from a non-cardiovascular cause are in competition. However, due to limitations in claim data, the cause of death could not be clearly identified, thus, competing risk was defined as a death without MACE in this study.

analysis could not be performed in this study. Additionally, this study used claim data from HIRA and NHIS, and it was not possible to collect exact laboratory data, including hemoglobin A1c, serum creatinine, and serum levels of each immunosuppressant. Therefore, we had no choice but to use operational definitions for PTDM, all clinical outcomes, and criteria for diabetes control in subgroup analyses. Above all, the possibility of over or under diagnosed PTDM should be considered when interpreting the results of this study. Lastly, we conducted a risk factor analysis including well-known risk factors for PTDM, such as infection with hepatitis C virus or cytomegalovirus; however, there are some limitations to determining risks precisely with an operational definition.

In conclusion, we found that PTDM is frequently observed in KT recipients and was associated with unfavorable outcomes, including MACEs and DCGF, in this Korean population-based study. By better understanding the risk variables, a dynamic approach to the surveillance and attenuation of transplant-related hyperglycemia may assist in diminishing negative patient and graft outcomes associated with PTDM. Thus, further studies with laboratory data and electronic medical records may reveal the risk factors and clinical outcomes of PTDM more precisely.

## Supplementary Information

Supplementary Information.

## Data Availability

The data that support the findings of this study are available from the corresponding author upon reasonable request.
