# Supplementary material for: Clinical outcomes of posttransplantation diabetes mellitus in kidney transplantation recipients: a nationwide population-based cohort study in Korea
Source: Sci Rep. 2022 Dec 14;12:21632. doi: 10.1038/s41598-022-25070-z (PMC9751267; doi:10.1038/s41598-022-25070-z)
Supplement: Supplementary file 1 — Supplementary Information. [file 41598_2022_25070_MOESM1_ESM.docx]

**Supplementary Materials**

**Figure S1.** Distribution of prescribed diabetes medications before and after kidney transplantation**.**

Others include SGLT-2 inhibitors, GLP-1agonists, and thiazolidinedione. *Abbreviations*: DM, diabetes mellitus; SGLT-2, Sodium-glucose transport protein 2; GLP-1, Glucagon-like peptide 1; DPP-4, Dipeptidyl peptidase-4

**
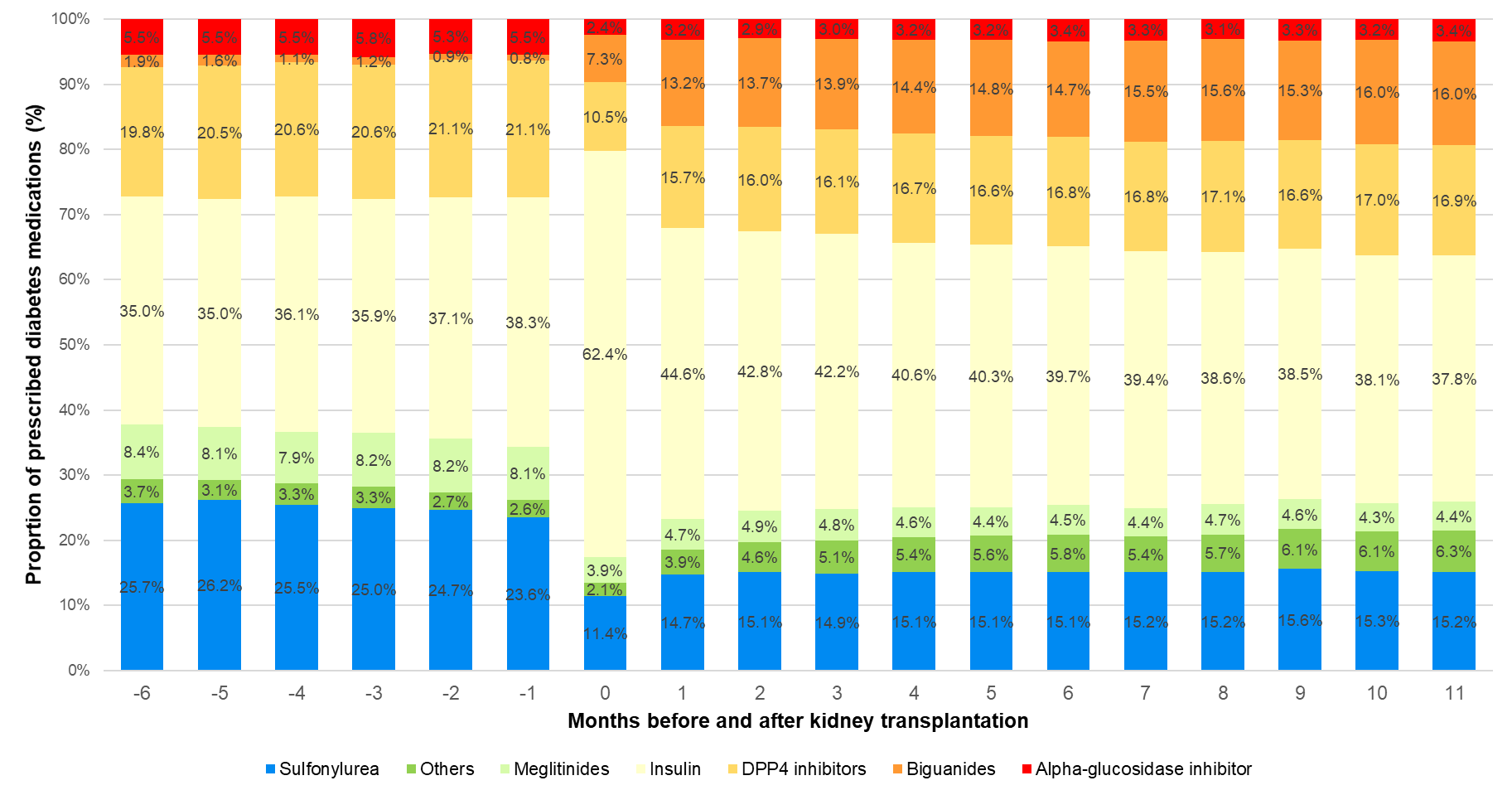
**

**Table S1.** Subgroup analyses in KT recipients according to the number of concomitant oral anti-diabetic medications

| **Outcomes** | **Total**  **(N)** | **Event**  **(N)** | **Person-**  **years** | **Incidence**  **rate** | **Model 1** | | **Model 2** | | **Model 3** | |
| --- | --- | --- | --- | --- | --- | --- | --- | --- | --- | --- |
|  |  |  |  |  | **HR (95% CI)** | ***P*** | **HR (95% CI)** | ***P*** | **HR (95% CI)** | ***P*** |
| **DCGF** |  |  |  |  |  |  |  |  |  |  |
| 1 | 385 | 19 | 1532 | 12.4 | Reference |  | Reference |  | Reference |  |
| 2 | 296 | 9 | 1248 | 7.2 | 0.57 (0.26–1.27) | 0.168 | 0.62 (0.27–1.39) | 0.244 | 0.60 (0.27–1.37) | 0.225 |
| ≥ 3 | 323 | 11 | 1618 | 6.8 | 0.54 (0.26–1.13) | 0.101 | 0.58 (0.27–1.24) | 0.158 | 0.57 (0.26–1.25) | 0.159 |
| **DWGF** |  |  |  |  |  |  |  |  |  |  |
| 1 | 385 | 4 | 1597 | 2.5 | Reference |  | Reference |  | Reference |  |
| 2 | 296 | 4 | 1285 | 3.1 | 1.26 (0.31–5.03) | 0.746 | 0.78 (0.17–3.51) | 0.742 | 0.79 (0.13–4.68) | 0.796 |
| ≥ 3 | 323 | 3 | 1649 | 1.8 | 0.72 (0.16–3.22) | 0.668 | 0.58 (0.12–2.78) | 0.500 | 0.59 (0.11–3.06) | 0.530 |
| **MACE** |  |  |  |  |  |  |  |  |  |  |
| 1 | 385 | 10 | 1583 | 6.3 | Reference |  | Reference |  | Reference |  |
| 2 | 296 | 8 | 1255 | 6.4 | 1.02 (0.40–2.58) | 0.969 | 0.80 (0.31–2.10) | 0.655 | 0.89 (0.33–2.39) | 0.812 |
| ≥ 3 | 323 | 10 | 1626 | 6.2 | 0.96 (0.40–2.31) | 0.931 | 0.89 (0.36–2.18) | 0.798 | 0.99 (0.40–2.49) | 0.990 |
| **All-cause mortality** |  |  |  |  |  |  |  |  |  |  |
| 1 | 385 | 5 | 1597 | 3.1 | Reference |  | Reference |  | Reference |  |
| 2 | 296 | 5 | 1285 | 3.9 | 1.26 (0.36–4.35) | 0.717 | 0.75 (0.19–2.92) | 0.678 | 0.80 (0.16–4.03) | 0.786 |
| ≥ 3 | 323 | 3 | 1649 | 1.8 | 0.57 (0.14–2.4) | 0.447 | 0.46 (0.10–2.07) | 0.312 | 0.48 (0.09–2.43) | 0.374 |

Model 1: Univariate analysis

Model 2: Model 1 + adjustment with age, sex, history of hypertension and dyslipidemia, dialysis modality, duration of dialysis

Model 3: Model 2 + adjustment with induction therapy, desensitization, rejection, use of steroid

*Abbreviations*: N, number; HR, hazard ratio; CI, confidence interval; DCGF, death-censored graft failure; DWGF, death with graft function; MACE, major adverse cardiovascular events

**Table S2.** Subgroup analyses in KT recipients according to the time of diagnosis of NODAT

| **Outcomes** | **Total**  **(N)** | **Event**  **(N)** | **Person-**  **years** | **Incidence**  **rate** | **Model 1** | | **Model 2** | | **Model 3** | |
| --- | --- | --- | --- | --- | --- | --- | --- | --- | --- | --- |
|  |  |  |  |  | **HR (95% CI)** | ***P*** | **HR (95% CI)** | ***P*** | **HR (95% CI)** | ***P*** |
| **DCGF** |  |  |  |  |  |  |  |  |  |  |
| < 6 months | 974 | 90 | 6046 | 14.89 | Reference |  | Reference |  | Reference |  |
| 6 months ~ 2 years | 337 | 32 | 1701 | 18.81 | 1.28 (0.86–1.93) | 0.227 | 1.20 (0.80–1.80) | 0.328 | 1.20 (0.80–1.81) | 0.386 |
| ≥ 2 years | 567 | 32 | 1435 | 22.3 | 1.57 (1.03–2.37) | 0.035 | 1.39 (0.91–2.14) | 0.129 | 1.45 (0.94–2.23) | 0.093 |
| **DWGF** |  |  |  |  |  |  |  |  |  |  |
| < 6 months | 974 | 41 | 6394 | 6.412 | Reference |  | Reference |  | Reference |  |
| 6 months ~ 2 years | 337 | 13 | 1805 | 7.201 | 1.07 (0.57–2.00) | 0.827 | 1.18 (0.63–2.21) | 0.600 | 1.16 (0.62–2.18) | 0.645 |
| ≥ 2 years | 567 | 10 | 1520 | 6.578 | 0.92 (0.46–1.86) | 0.820 | 1.20 (0.59–2.45) | 0.612 | 1.25 (0.61–2.56) | 0.544 |
| **MACE** |  |  |  |  |  |  |  |  |  |  |
| < 6 months | 974 | 49 | 6217 | 7.881 | Reference |  | Reference |  | Reference |  |
| 6 months ~ 2 years | 337 | 27 | 1749 | 15.44 | 1.98 (1.24–3.18) | 0.005 | 2.13 (1.33–3.43) | 0.002 | 2.31 (1.32–2.43) | 0.440 |
| ≥ 2 years | 567 | 15 | 1491 | 10.06 | 1.32 (0.73–2.39) | 0.363 | 1.64 (0.90–3.02) | 0.109 | 1.68 (0.91–3.09) | 0.095 |
| **All-cause mortality** |  |  |  |  |  |  |  |  |  |  |
| < 6 months | 974 | 46 | 6394 | 7.194 | Reference |  | Reference |  | Reference |  |
| 6 months ~ 2 years | 337 | 17 | 1805 | 9.417 | 1.27 (0.73–2.21) | 0.404 | 1.37 (0.78–2.40) | 0.268 | 1.35 (0.77–2.37) | 0.295 |
| ≥ 2 years | 567 | 10 | 1520 | 6.578 | 0.87 (0.44–1.75) | 0.706 | 1.09 (0.54–2.22) | 0.802 | 1.12 (0.55–2.29) | 0.748 |

Model 1: Univariate analysis

Model 2: Model 1 + adjustment with age, sex, history of hypertension and dyslipidemia, dialysis modality, duration of dialysis

Model 3: Model 2 + adjustment with induction therapy, desensitization, rejection, use of steroid

*Abbreviations*: HR, hazard ratio; CI, confidence interval; DCGF, death-censored graft failure; DWGF, death with graft function; MACE, major adverse cardiovascular events; NODAT, New onset diabetes after kidney transplantation

**Table S3.** Subgroup analyses in KT recipients according to whether stop prescribing anti-diabetic medications^a^

| **Outcomes** | **Total**  **(N)** | **Event**  **(N)** | **Person-**  **years** | **Incidence**  **rate** | **Model 1** | | **Model 2** | | **Model 3** | |
| --- | --- | --- | --- | --- | --- | --- | --- | --- | --- | --- |
|  |  |  |  |  | **HR (95% CI)** | ***P*** | **HR (95% CI)** | ***P*** | **HR (95% CI)** | ***P*** |
| **DCGF** |  |  |  |  |  |  |  |  |  |  |
| Keep | 1399 | 105 | 6109.2 | 17.2 | Reference |  | Reference |  | Reference |  |
| Stop | 474 | 45 | 3072.5 | 14.7 | 1.04 (0.73–1.49) | 0.820 | 0.98 (0.68–1.41) | 0.904 | 0.95 (0.66–1.36) | 0.768 |
| **DWGF** |  |  |  |  |  |  |  |  |  |  |
| Keep | 1361 | 54 | 6179.9 | 8.7 | Reference |  | Reference |  | Reference |  |
| Stop | 516 | 9 | 3539.8 | 2.5 | 0.42 (0.21–0.85) | 0.016 | 0.45 (0.22–0.91) | 0.026 | 0.43 (0.21-0.87) | 0.018 |
| **MACE** |  |  |  |  |  |  |  |  |  |  |
| Keep | 1365 | 73 | 5998 | 12.2 | Reference |  | Reference |  | Reference |  |
| Stop | 512 | 18 | 3459.7 | 5.2 | 0.58 (0.33–1.01) | 0.055 | 0.66 (0.38–1.14) | 0.136 | 0.68 (0.39–1.17) | 0.164 |
| **All-cause mortality** |  |  |  |  |  |  |  |  |  |  |
| Keep | 1361 | 57 | 6179.9 | 9.2 | Reference |  | Reference |  | Reference |  |
| Stop | 516 | 15 | 3539.8 | 4.2 | 0.64 (0.37–1.11) | 0.115 | 0.68 (0.38–1.18) | 0.169 | 0.66 (0.37–1.15) | 0.142 |

^a^These subgroup analyses were conducted except when each clinical outcome occurred at the time of diagnosis of NODAT

Model 1: Univariate analysis

Model 2: Model 1 + adjustment with age, sex, history of hypertension and dyslipidemia, dialysis modality, duration of dialysis

Model 3: Model 2 + adjustment with induction therapy, desensitization, rejection, use of steroid

*Abbreviations*: N, number; HR, hazard ratio; CI, confidence interval; DCGF, death-censored graft failure; DWGF, death with graft function; MACE, major adverse cardiovascular events

**Table S4.** Baseline characteristics of total enrolled participants according to PTDM after propensity score matching

| **Variables** | | **PTDM (-) (n = 1878)** | **PTDM (+) (n = 1878)** | ***P*** |
| --- | --- | --- | --- | --- |
| **Age, years** |  | 49.3 ± 10.8 | 49.6 ± 10.7 | 0.320 |
|  | ≤30 years | 97 (5.2%) | 97 (5.2%) | 0.998 |
|  | 31 – 40 years | 253 (13.5%) | 256 (13.6%) |  |
|  | 41 – 50 years | 557 (29.7%) | 560 (29.8%) |  |
|  | 51 – 60 years | 704 (37.5%) | 694 (37.0%) |  |
|  | >60 years | 267 (14.2%) | 271 (14.4%) |  |
| **Gender, male, n (%)** |  | 1133 (60.3%) | 1120 (59.6%) | 0.689 |
| **Dialysis modality** |  |  |  | 0.719 |
|  | Hemodialysis | 889 (47.3%) | 885 (47.1%) |  |
|  | Peritoneal dialysis | 330 (17.6%) | 315 (16.8%) |  |
|  | Mixed | 82 (4.4%) | 95 (5.1%) |  |
|  | Preemptive | 577 (30.7%) | 583 (31.0%) |  |
| **Dialysis duration (years)** |  |  |  | 0.914 |
|  | None | 183 (9.7%) | 186 (9.9%) |  |
|  | < 1 years | 541 (28.8%) | 540 (28.8%) |  |
|  | 1 years – 2 years | 279 (14.9%) | 271 (14.4%) |  |
|  | 2 years – 3 years | 216 (11.5%) | 234 (12.5%) |  |
|  | ≥3 years | 659 (35.1%) | 647 (34.5%) |  |
| **Underlying disease** |  |  |  |  |
|  | Hypertension | 1805 (96.1%) | 1799 (95.8%) | 0.679 |
|  | Dyslipidemia | 1070 (57.0%) | 1064 (56.7%) | 0.869 |
|  | Cardiovascular disease | 366 (19.5%) | 383 (20.4%) | 0.514 |
| **Induction therapy** |  |  |  | 0.007 |
|  | None | 47 (2.5%) | 36 (1.9%) |  |
|  | Thymoglobulin | 235 (12.5%) | 180 (9.6%) |  |
|  | Basiliximab | 1536 (81.8%) | 1583 (84.3%) |  |
|  | Both | 60 (3.2%) | 79 (4.2%) |  |
| **Maintenance immunosuppression** | |  |  |  |
|  | Tacrolimus | 1370 (72.9%) | 1344 (71.6%) | 0.362 |
|  | Cyclosporine | 165 (8.8%) | 152 (8.1%) | 0.481 |
|  | MMF | 1265 (67.4%) | 1220 (65.0%) | 0.129 |
|  | Steroid | 1417 (75.5%) | 1421 (75.7%) | 0.909 |
| **Rejection** |  | 208 (11.1%) | 265 (14.1%) | 0.006 |
|  | High dose steroid | 194 (10.3%) | 238 (12.7%) | 0.008 |

*Abbreviations*: PTDM, New onset diabetes after kidney transplantation; MMF, mycophenolate mofetil

**Table S5.** Incidence rate and hazard ratio of adverse outcomes according to PTDM after propensity score matching

| **Outcomes** | **PTDM** | **N** | **Cases** | **Person-years** | **Incidence rate** | **Model 1** | | **Model 2** | | **Model 3** | |
| --- | --- | --- | --- | --- | --- | --- | --- | --- | --- | --- | --- |
|  |  |  |  |  |  | **HR (95% CI)** | ***P*** | **HR (95% CI)** | ***P*** | **HR (95% CI)** | ***P*** |
| **DCGF** |  |  |  |  |  |  |  |  |  |  |  |
|  | PTDM (-) | 1878 | 120 | 12029.5 | 10.0 | Reference | | Reference | | Reference | |
|  | PTDM (+) | 1878 | 154 | 12633.1 | 12.2 | 1.80 (1.42-2.30) | <0.001 | 1.86 (1.45-2.38) | <0.001 | 1.83 (1.42-2.35) | <0.001 |
| **DWGF** |  |  |  |  |  |  |  |  |  |  |  |
|  | PTDM (-) | 1878 | 71 | 12420.4 | 5.7 | Reference | | Reference | | Reference | |
|  | PTDM (+) | 1878 | 64 | 13171.0 | 4.9 | 1.36 (0.96-1.91) | 0.083 | 1.23 (0.88-1.73) | 0.233 | 1.17 (0.84-1.65) | 0.356 |
| **MACE** |  |  |  |  |  |  |  |  |  |  |  |
|  | PTDM (-) | 1878 | 76 | 12161.2 | 6.2 | Reference | | Reference | | Reference | |
|  | PTDM (+) | 1878 | 91 | 12909.1 | 7.1 | 1.93 (1.41-2.65) | <0.001 | 1.76 (1.28-2.43) | 0.001 | 1.79 (1.30-2.46) | <0.001 |
| **All–cause mortality** |  |  |  |  |  |  |  |  |  |  |  |
|  | PTDM (-) | 1878 | 82 | 12420.4 | 6.6 | Reference | | Reference | | Reference | |
|  | PTDM (+) | 1878 | 73 | 13171 | 5.5 | 1.31 (0.95-1.81) | 0.096 | 1.20 (0.87-1.65) | 0.268 | 1.15 (0.84-1.59) | 0.387 |

Model 1: Univariate analysis

Model 2: Model 1 + adjustment with age, sex, history of hypertension, dyslipidemia, and cardiovascular disease, dialysis modality, duration of dialysis

Model 3: Model 2 + adjustment with induction therapy, desensitization, rejection, use of steroid

*Abbreviations*: PTDM, New onset diabetes after kidney transplantation; N, number; HR, hazard ratio; CI, confidence interval; DCGF, death-censored graft failure; DWGF, death with graft function; MACE, major adverse cardiovascular events
